# Cholecystomegaly: laparoscopic treatment

**DOI:** 10.4103/0972-9941.16534

**Published:** 2005-06

**Authors:** D. J. Tessier, L. McMahon, K. L. Harold

**Affiliations:** Department of Surgery, Mayo Clinic Scottsdale, Scottsdale, AZ, USA

**Figure 1:**
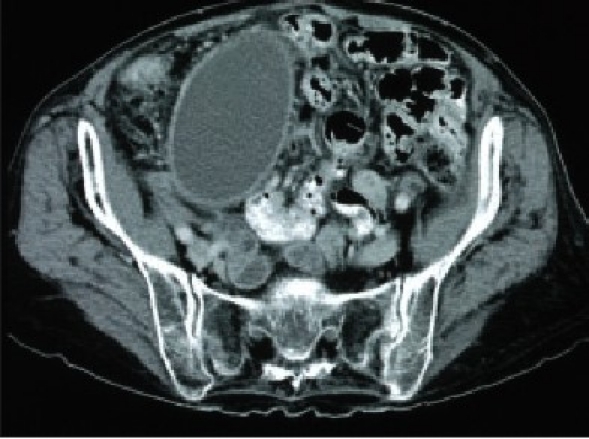
Computerized tomography (CT) scan demonstrating massive gallbladder extending to the pelvic brim

**Figure 2:**
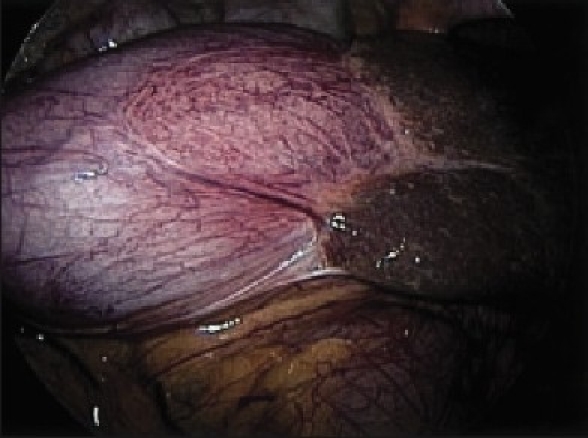
Laparoscopic view demonstrating an enlarged gallbladder

A 78-year-old patient was noted to be severely anemic (Hb 4.9) during a hospital admission in August 2002 for lightheadedness. She underwent a thorough workup including an abdominal computed tomography (CT) scan which demonstrated a mildly dilated gallbladder and possible pancreatic mass. Endoscopic retrograde cholangiopancreaticography (ERCP) was performed which showed a mildly dilated common bile duct without filling defects. No abnormalities were identified in the pancreas. During ERCP it was noted that the patient had a large hiatal hernia with Cameron's lesions. She subsequently underwent a laparoscopic fundoplication with resolution of her anemia. The patient did well for 16 months until she presented to her primary care physician with back pain. On physical examination she had a large abdominal mass. She underwent a CT scan that demonstrated marked enlargement of her gallbladder extending below her pelvic brim [Figure 1]. Her liver function tests were normal. MRCP demonstrated normal pancreatic and common bile ducts along with the dilated gallbladder. Due to the large size of the gallbladder and history of ductal dilatation, laparoscopic cholecystectomy was recommended. At the time of operation the gallbladder was found to be massively dilated with a thickened wall [Figure 2]. Needle decompression (with extraction of 200 cm^3^ of bile) was used to decrease the gallbladder size and make it manageable. The gallbladder was successfully removed laparoscopically using a standard dissection and port placement. The specimen was removed through the umbilical port, which required slight enlargement for extraction. Intraoperative cholangiogram showed normal bile duct size without filling defects. The specimen measured 17 cm × 8 cm × 2 cm. Pathology showed impaction of a stone in the neck of the gallbladder, explaining the dilatation, as well as acute and chronic cholecystitis. At 6-month follow up the patient is doing well and her back pain has resolved.

Since its introduction, laparoscopic cholecystectomy has been the preferred treatment of symptomatic cholelithiasis.[1] Relative contraindications include cirrhosis, coagulopathy, pancreatitis, pregnancy, morbid obesity, and severe cardiorespiratory insufficiency.[2] A theoretical risk with a massively dilated gallbladder is volvulus. Gallbladder volvulus is a rare event with less that 400 cases reported in the literature to date.[3] Volvulus commonly occurs in elderly women with visceroptosis. There are two known anatomic conditions that predispose a patient to volvulus. A gallbladder that is not anchored to the liver bed and only has attachment via the cystic artery and duct is at risk for torsion. Additionally, a gallbladder with a very long mesentery, as can be seen with long-standing massive dilatation, may also undergo volvulus. Unfortunately, neither of these conditions can be definitively diagnosed preoperatively to determine who is at risk. Given the massive size of the gallbladder in our patient we felt laparoscopic cholecystectomy was warranted. This case demonstrates the ability to safely perform laparoscopic cholecystectomy even in the setting of a massively dilated gallbladder. Additionally, it highlights the possibility of an impacted stone when a dilated gallbladder is encountered, even in a chronic setting. A preoperative ultrasound may have diagnosed the impacted gallstone compared to CT.
